# COVID-19 as a Trigger of Recurrent Guillain–Barré Syndrome

**DOI:** 10.3390/pathogens9110965

**Published:** 2020-11-19

**Authors:** Erin P. McDonnell, Nicole J. Altomare, Yesha H. Parekh, Ram C. Gowda, Payal D. Parikh, Mark H. Lazar, Martin J. Blaser

**Affiliations:** 1 Department of Medicine, Rutgers Robert Wood Johnson Medical School, New Brunswick, NJ 08901, USA; erin.mcdonnell@rutgers.edu (E.P.M.); nja71@rwjms.rutgers.edu (N.J.A.); yhp7@rwjms.rutgers.edu (Y.H.P.); parikhpd@rwjms.rutgers.edu (P.D.P.); 2 Department of Neurology, Rutgers Robert Wood Johnson Medical School, New Brunswick, NJ 08901, USA; rg921@rwjms.rutgers.edu (R.C.G.); marklaz@gmail.com (M.H.L.); 3 Center for Advanced Biotechnology and Medicine, Rutgers University, Piscataway, NJ 08854, USA

**Keywords:** SARS-CoV-2 neurological complications, recurrent Guillain–Barré syndrome, para-infectious Guillain–Barré syndrome, Chronic Inflammatory Demyelinating Neuropathy, demyelinating polyradiculopathies

## Abstract

Coronavirus 2019 (COVID-19) has been reported to trigger Guillain–Barré syndrome (GBS). While uncommon, recurrent GBS (rGBS) episodes, triggered by antecedent viral infections, have been reported in a small proportion of GBS patients, here we describe a patient with a recurrent case of GBS, occurring secondary to COVID-19 infection. Before this patient’s episode, he had two prior GBS flares, each precipitated by a viral infection followed by complete recovery besides intermittent paresthesias. We also consider the nosology of this illness in the spectrum of rGBS and Chronic Inflammatory Demyelinating Polyneuropathy (CIDP), with their differing natural histories, prognosis, and therapeutic approaches. For patients who have a history of inflammatory demyelinating polyradiculopathies who develop COVID-19, we recommend close observation for neurologic symptoms over the next days and weeks.

## 1. Introduction

The novel coronavirus disease 2019 (COVID-19), caused by severe acute respiratory syndrome coronavirus 2 (SARS-CoV-2), was first documented in December 2019 in Wuhan, China. While COVID-19 predominantly leads to respiratory symptoms, several neurological symptoms have also been reported, including headache, syncope, myalgia, anosmia, and ageusia [1,2,3]. Recent research has shown that COVID-19 has been associated with a broad range of immune neuropathies, including Guillain–Barré syndrome (GBS) and the worsening of Chronic Inflammatory Demyelinating Polyradiculopathy (CIDP) [4,5]. GBS is an acute monophasic demyelinating polyradiculopathy, presenting with a spectrum of limb and cranial nerve weaknesses, sensory symptoms, and autonomic dysfunction [6,7]. Here, we describe the first case, to our knowledge, of recurrent GBS (rGBS) secondary to COVID-19 infection.

rGBS is defined as two or more GBS episodes that have either ≥4 months between episodes without full recovery, or ≥2 months between episodes if the patient exhibits complete or near-complete recovery. rGBS occurs in 2–5% of patients with previously diagnosed GBS, and rGBS patients often have a preceding illness with rapid symptom onset; symptoms are often similar even with different viral triggers [8,9]. Diagnostic criteria for GBS include progressive motor weakness in at least one extremity, which can range from weakness to complete paralysis, and areflexia [10].

Rather than two distinct diagnoses, there may be a spectrum between rGBS and CIDP in which patients typically present with chronic mixed motor and sensory neuropathies following sub-acute onset, with relapses and remissions [10]. CIDP presents with symmetric weakness both distally and proximally that progressively worsens, though sometimes with a relapsing-remitting course. Motor deficits are the most problematic, while autonomic and pain symptoms are less common. The respiratory and facial muscles are rarely impacted. There are also arguments that diabetes mellitus is a risk factor for CIDP [11]. While this is a minority view, it is important to consider, given our patient’s diagnosis of Type 2 diabetes mellitus.

rGBS can be differentiated from CIDP by bulbar and respiratory muscle weakness and an antecedent infection before each GBS episode [8,9]. Another distinction between these diseases is the time from neurological symptoms to maximum disability. In rGBS this window is often less than 4 weeks, while CIDP by definition progresses over at least 2 months [8,12,13]. While many studies discuss these two diagnoses separately, rGBS and CIDP may represent a range of inflammatory demyelinating polyradiculopathies rather than distinct diagnoses [10]. We report the clinical presentation of a COVID-19 patient whose disease may lie on a spectrum between rGBS and CIDP.

## 2. Case Presentation 

In 2009, at age 43, the patient was in a motor vehicle accident and came to the Rutgers Robert Wood Johnson University Hospital (RWJUH) Emergency Department complaining of numbness and tingling in both upper extremities. He denied bowel or bladder dysfunction or focal weakness but had a positive straight leg test, decreased ankle jerk on the left, and patchy sensory loss in his upper extremities. He suffered injuries to the cervical and lumbosacral spine and was ultimately diagnosed with a herniated disc and discharged within one hour of presentation.

In 2013, at age 47, the patient’s past medical history was significant for Type 2 diabetes mellitus (T2DM) with elevated post-prandial glucose, along with a history of herniated nucleus pulposus at C6-C7, L2-3, L3-4, L4-L5 with disc bulges. In 2013, he developed an acute illness with cough, fever (maximum temperature of 39.2 °C), and ageusia (food tasted like clay), which was diagnosed as Influenza A. Three days later, he developed numbness in both arms and legs, left greater than right. The numbness progressed to his trunk area throughout the day, and he felt weak when walking up stairs. One day later, he noticed slurred speech, and his wife noticed left facial weakness. He sought medical attention from a neurologist at the Neurology and Headache Center of New Jersey. Work-up revealed an isolated elevation in cerebrospinal fluid (CSF) protein, but otherwise, laboratory tests showed no abnormalities (Table 1). The magnetic resonance imaging (MRI) of the brain, cervical, thoracic, and lumbar spine were unremarkable except for a mild multilevel cervical spondylosis, peripheral annular tear on L3-4 (left) and a midline annular tear at L4-5 with a small midline disk herniation. The physician’s differential diagnosis included GBS, multiple sclerosis, and Lyme disease. The patient’s neurological symptoms completely resolved at home within one week of presentation without any treatment, but he had occasional fleeting paresthesias typically on his hands, sometimes feet, and rarely his trunk.

In 2017, at age 51, the patient had influenza-like symptoms, including productive cough and ageusia again. Three days later, he developed numbness and tingling in both arms and legs, and weakness in his right leg, progressing over two days and returned to the same neurologist. Electromyography (EMG) revealed absent F waves in the right fibular and post tibial nerves. In the right fibular nerve, the motor nerve conduction velocity (NCV) was 26.7 m/s, representing a 36% decrease compared to the lower limit of normal, (41 m/s). There also was a > 70% decrease from the distal amplitude (7.1 mV) compared to the proximal amplitude (2.1 mV) of the compound motor unit action potential (CMAP) in the right median nerve. Dispersion was noted in the right median, ulnar, and fibular nerve CMAPs, and there was a decrease in the distal CMAP of both the right posterior tibial and right ulnar nerves. The right posterior tibial nerve showed a 77% decrease from 1.1 mV distally to 0.25 mV proximally. The right ulnar nerve showed a 62% decrease from 5.5 mV distally to 2.1 mV proximally. There were no technical confounders in the EMG test. These findings of decreased NCV, dispersion, decrease in the CMAP, and absent F waves all support our diagnosis of demyelinating neuropathy and are consistent with the criteria for both rGBS and CIDP [12,13].

The next day, he had increased right leg weakness, dysarthria, slurring of speech, trouble eating and manipulating his saliva, and the acute onset of facial droop. On admission to Robert Wood Johnson University Hospital (RWJUH), he reported that the symptoms he was experiencing were more severe than those in the 2013 episode. Neurological examination revealed intact cranial nerves except for an isolated left facial droop, 4/5 motor strength in right leg compared to 5/5 in left leg (using Medical Research Council Score) [14], mild stocking loss vibration sense otherwise intact to pin, light touch, and position sense, normal gait, and 2+ reflexes bilaterally. Laboratory tests were normal, but a respiratory viral panel (GenMark eSensor; Carlsbad CA), conducted on nasopharyngeal swabs using qualitative nucleic acid multiplex reverse transcriptase PCR amplification, was positive for Influenza B, and protein level in the CSF also was increased (Table 1 and Table 2). MRI with and without contrast of the brain and cervical spine were unremarkable except for mild multilevel cervical spondylosis and new C4-5 small central disk osteophyte complex contributing to mild spinal canal stenosis. On hospital day 1, the patient reported an isolated improvement in his dysarthria. After a repeat lumbar puncture revealed a mild increase in CSF protein, the patient was started on a seven-day treatment of Prednisone, 60 mg daily. On day 2, the patient’s left facial droop improved significantly and had improved leg strength and sensory deficient in his arms and legs. With his prior history of an acute self-resolving polyneuropathy/polyradiculitis episode in 2013 combined with the current episode following a symptomatic Influenza B infection, the differential diagnosis included rGBS and CIDP. He was discharged from the hospital after two days, and by two weeks had made a complete recovery with no residual deficits aside from occasional hand and foot paresthesias after showering.

After this second episode, the patient had a complete workup by a neurologist specializing in GBS. He was noted to consistently have a 50% conduction velocity deficit even when without neurologic symptoms. He was diagnosed with autoimmune polyneuropathy since he did not fit the diagnosis of either GBS or CIDP at the time. He was subsequently put on an autoimmune diet, which led to a decrease in his intermittent paresthesias.

In January 2020, the patient had a two-day illness with fevers, numbness in his hands and fingers, and ageusia. These symptoms resolved spontaneously, and he had no further sequelae. The patient was not tested for any infection and did not seek medical advice or treatment.

In April 2020, the patient, now 54-years old, presented to the emergency department with complaints of progressive dysphagia and difficulty in managing his secretions for two days. The patient reported being febrile three days before admission, followed one day later by progressive dysphagia, facial weakness, upper and lower extremity weakness leading to inability to move his legs, and paresthesia over his hands and feet. He was admitted to the neurologic intensive care unit for close monitoring. Upon initial evaluation, the patient was febrile to 38.1 °C and had facial diplegia with trace eyebrow raise and smile, decreased sensation to light touch over the lower face, difficulty swallowing and extending his tongue, and moderate dysarthria. Strength in his upper extremities was 4/5 bilaterally, and his lower extremity hip flexion was 3/5 bilaterally, all weaker proximally than distally. The patient had decreased sensation to light touch in a stocking-glove distribution. Deep tendon reflexes were +1 throughout. Chest x-ray (CXR) on admission showed no evidence of acute cardiopulmonary abnormalities. A lumbar puncture on admission showed no evidence of oligoclonal bands but was significant for isolated CSF protein elevation (albuminocytologic dissociation) (Table 1). Reverse transcription-polymerase chain reaction (RT-PCR) test for COVID-19 was positive on hospital day 1. Tests for Lyme disease, human immunodeficiency virus, viral hepatitis, anti-nuclear antibody, rheumatoid factor, and anti-GM1 ganglioside IgG and IgM were all negative.

He was diagnosed with and managed for rGBS secondary to COVID-19 viral illness. He received intravenous immunoglobulin (IVIg, 0.4 g/kg) on hospital day 1, continuing for 5 days, and 400 mg of hydroxychloroquine (HCQ) on hospital days 4–8, according to standard COVID-19 treatment at that time. Oxygen saturation remained > 90% on ambient air for the first six days in the hospital, but on day 7, he required supplemental oxygen (nasal cannula at two liters); repeat CXR showed new bilateral pulmonary infiltrates. A retest for COVID-19 on hospital day 6 was again positive. During his hospitalization, he could lift his arms but had no hand strength and abdominal muscle weakness that prevented him from changing his position. He experienced facial and lip numbness and was unable to control his tongue, which led to apneic episodes when it slid back into his mouth. Throughout the patient’s hospital stay, he experienced right epistaxis secondary to his nasogastric tube, elevated transaminases due to HCQ or COVID-19, as well as urinary retention for which an. An indwelling urinary catheter was placed on day 3 for urinary retention. He passed a voiding trial on day 8 of admission, with subsequent removal of the catheter, as well as a swallowing evaluation on day 10. With improving muscle strength, the patient was discharged on day 11.

Post-discharge, the patient had residual weakness for five days and had continued desaturation (to 89%) after removing supplemental oxygen. By nine days post-discharge, the muscle weakness had resolved, but the patient continued to have nocturnal dyspnea. Two weeks after discharge, the patient was asymptomatic and no longer required supplemental oxygen. In contrast to his prior flares in which the GBS-related symptoms resolved quickly, the GBS-related symptoms from this episode persisted for over one week, with residual weakness for an additional week after discharge. The patient reports full recovery from muscle weakness with full muscle strength and returned to normal rigorous exercise and endurance training. However, five months post-discharge, he reported severe persistent numbness of the medial left knee up to the medial thigh. It is unclear whether these symptoms are due to an unrelated peripheral mononeuropathy, radiculopathy, or represent residual symptoms from his most recent GBS episode. The patient reported that in total, the COVID-19 GBS flare resulted in more severe symptoms and a longer recovery time compared to his previous GBS episodes.

## 3. Discussion

We report a patient with rGBS whose most recent flare was triggered by antecedent COVID-19 infection. COVID-19, generally considered a respiratory infection, may lead to neurological conditions, including encephalitis, stroke, acute disseminated encephalomyelitis, and peripheral neuropathies, such as GBS [15,16]. Since rGBS patients represent 2–5% of patients with GBS, characterizing the manifestations of rGBS flares after COVID-19 may improve diagnostic and therapeutic approaches [8,9].

The time interval from acute viral illnesses to GBS symptom onset is typically several days to a few weeks. Although it may not be typical, a short interval between febrile illness onset to symptom worsening has been reported [5,17,18]. The reported cases are patients with COVID-19, which suggests that this new disease may have a different spectrum of illness than usually seen. Based on the Asbury et al. [19] clinical findings required to diagnose GBS, our patient had motor weakness progression occurring within four weeks after the trigger illness, symmetry between the affected limbs, mild sensory symptoms, cranial nerve involvement, recovery of symptoms within two to four weeks after the motor weakness progression stopped, and signs of autonomic dysfunction; however, he lacked the typical areflexia. Clinical features and neurologic signs that lessen the probability of GBS include motor weakness asymmetry between the affected limbs, continuous bladder or bowel dysfunction, CSF pleocytosis, and a distinct sensory level [19].

**Figure 1:**
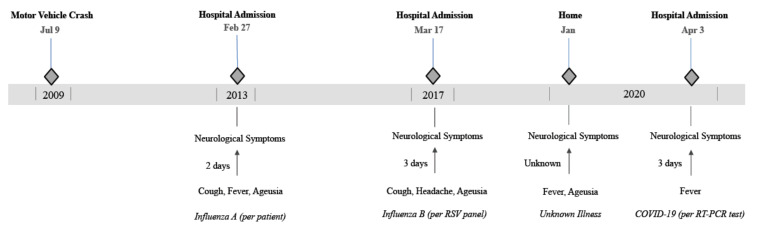
Timeline of events related to recurrent neurological symptoms. The figure highlights the antecedent viral infection, if known, the interval from flu-like symptoms to neurological symptoms as well as the decreasing interval between flares.

Patients with rGBS generally have the same signs and symptoms with each flare, regardless of the triggering infection, suggesting a common pathway of nerve injury after varied infectious and immunological insults. The GBS clinical presentation also can vary due to host immunologic genotype. In previously reported rGBS cases, the intervals between flares decreased, while the episode severity and CSF protein levels increased [9], findings all observed in our patient (Table 1 and Table 2, Figure 1). Based on these criteria, the patient’s clinical presentation and physical exam findings suggest rGBS as a likely diagnosis (Table 2).

In contrast, a definitive CIDP diagnosis requires both of the following: progressive, stepwise, recurrent proximal or distal weakness, and sensory dysfunction that develops over the course of two months, as well as areflexia or hyporeflexia [13]. Supportive findings overlap with GBS and include CSF albuminocytologic dissociation in each episode, MRI enhancement of nerve roots or plexi, nerve conduction velocities < 80% of the lower limit of normal, clinical improvement following immunomodulation, and demyelination on nerve biopsy. The electrodiagnostic criteria for CIDP include absent F waves in two nerves, especially if they have a distal negative peak, compound muscle action potential > 20% the lower limit of normal [13]. The patient’s 2017 episode meets the CIDP electrodiagnostic criteria (Table 3).

There is evidence that CIDP and diabetic neuropathy may be related to CIDP [11]. In one study, CIDP prevalence was nine times higher in patients with Type 2 diabetes (T2D) than those without [20]. The presentations of T2D-related CIDP and idiopathic CIDP are similar but cannot be easily distinguished without additional testing. The summative nerve damage accompanying diabetes and CIDP together cause more severe axonal loss [13]. However, this explanation is unlikely for our patient due to the episodic nature of the disease, the CSF findings, and the axonal preservation indicated by the 2017 EMG results. There is also evidence that the patient’s neurological symptoms are not a misdiagnosed diabetic neuropathy, including that the patient’s symptoms are episodic and transient, which are atypical in diabetic neuropathy, and also that the patient’s diabetes is well-controlled. Of note, the patient’s serum glucose levels during his inflammatory states ranged from 109 to 136 mg/dL. While our patient carries the diagnosis of diabetes mellitus, he was not prescribed any medication and his glucose levels were well-controlled with diet. The inflammatory state of COVID-19, considered an example of “stress hyperglycemia” [21], and our patient’s minor glucose elevations are consistent with that. His essentially conserved glucose control makes diabetes mellitus a less likely contributor to his demyelinating neuropathies compared to rGBS/CIDP.

Since the patient meets the criteria for both rGBS and CIDP (Table 3), his presentation is consistent with the hypothesis that these two diagnoses exist on a continuum rather than being distinct [9]. Along that continuum, this patient might be classified as closer to rGBS, with 3–4 years between symptom flares that include bulbar signs, each triggered by an antecedent viral infection, complete resolution of neurological symptoms (besides intermittent paresthesia), and albuminocytologic dissociation in the CSF. In addition, GBS patients typically have CSF protein levels ranging from 54–100 mg/dL [22]. However, CSF protein levels typically do not peak until a few weeks after the onset of illness [22]. Our patient’s lumbar puncture was done within a week of the onset of his neurological symptoms yet was within the GBS range. Favoring CIDP is the potential diagnosis of T2DM, albuminocytologic dissociation, and CIDP-consistent EMG result (demyelinating neuropathy without axonal damage), with flares secondary to antecedent viral infections [13,23,24]. The lack of EMG results and nerve biopsy from the most recent GBS episode following SARS-CoV-2 infection is limiting.

The distinction between rGBS and CIDP is important since the treatments differ. Short-term IVIg or plasma exchange is usually used for GBS or rGBS, while CIDP patients require long-term treatment with IVIg, steroids, plasma exchange, or immunosuppressive therapy [9,10,25,26]. Our patient received no treatment in 2013, a seven-day steroid course in 2017, and five-days of IVIg in 2020, consistent with his worsening episodes. Although the patient improved shortly after receiving corticosteroids in 2017, consistent with CIDP, the episodic nature of his illness, requiring only short-term therapy, favors rGBS.

## 4. Conclusions

While initial GBS episodes triggered by COVID-19 have been well-reported, we describe the first case of rGBS to our knowledge and show that its severity was enhanced compared to prior episodes. This case report also adds to the scarce literature that has already proposed a spectrum of relapsing-remitting demyelinating disorders [10]. Overall, better disease nosology improves proper diagnosis and treatment. The growing body of research will likely show that the rate of GBS in COVID-19 patients is higher than the background GBS incidence. Studying rGBS patients allows us to understand COVID-19 pathophysiology better and for comparisons with other viral antecedents to GBS. For patients who have a history of inflammatory demyelinating polyradiculopathies who develop COVID-19, we recommend close observation for neurologic symptoms over the next days and weeks.

## Figures and Tables

**Table 1:** Pertinent laboratory findings for each Guillain–Barré syndrome (GBS) episode.

	Reference Values	2013	2017	2020
CSF protein (mg/dL)	15–45	58	63	74
CSF glucose (mg/dL)	40–80	73	73	69
CSF RBC Count	0	0	49	2
CSF Polysegmented Neutrophils	0	0	0	0
Serum glucose (mg/dL)	70–100	115	136	109
C-Reactive Protein (mg/dL)	0.00–0.70			0.98
Respiratory Viral Panel			Influenza B	

**Table 2:** Summary of hospital admissions: illness onset, initial presentation, and illness severity.

	2009	2013	2017	2020
Chief Complaint	Numbness in upper extremities and groin pain	Mild weakness in leg, numbness, slurred speech, and ageusia	Dysarthria, trouble eating and manipulating saliva, and ageusia	Dysarthria, trouble eating, weakness in legs
Interval from non-neurologic initial symptoms to onset of neurological symptoms	N/A—Motor Vehicle Crash	2 days	3 days	3 days
Initial symptoms	N/A	Cough, chills, fever (39.2°C)	Cough, headache	Fever (38.1°C)
Cranial Nerve Assessment	N/A	Intact.	Left facial droop.	Progressive dysphagia, facial weakness, facial diplegia, decreased sensation to touch over lower face, difficulty protracting tongue, and moderate dysarthria.
Cranial Nerve Assessment	N/A	Intact.	Left facial droop.	Progressive dysphagia, facial weakness, facial diplegia, decreased sensation to touch over lower face, difficulty protracting tongue, and moderate dysarthria.
Sensory Nerve Findings on Physical Exam	Decreased light touch, sensation, and vibration in upper extremities with the thumb and index finger most affected.		Mild stocking loss vibration sense otherwise intact to pin, light touch, and position sense.	Decreased sensation to light touch in glove-and-stocking distribution.
Motor Nerve Findings on Physical Exam	Normal gait. Reflexes 2+ bilaterally, except for 1+ left ankle.	Normal gait. Strength 5/5 bilaterally in upper and lower extremities except 4/5 right hip abductors.	Normal gait. 4/5 strength right leg Reflexes 2+ bilaterally.	4/5 strength in bilateral upper extremities with increased weakness proximally. 3/5 strength in bilateral lower extremity hip flexion with increased weakness proximally. Reflexes 1+ bilaterally.
Suspected GBS Trigger	N/A	Influenza A (per patient)	Influenza B (per RSV panel)	COVID-19 (per RT-PCR test)
Length of Hospital Stay	< 1 day	Out-patient	2 days	11 days

**Table 3:** Assessment of patient’s symptoms based on recurrent Guillain–Barré syndrome (rGBS) and Chronic Inflammatory Demyelinating Polyneuropathy (CIDP) diagnostic criteria [6,7,8,9,10,16,17].

rGBS Diagnostic Criteria	CIDP Diagnostic Criteria
Viral trigger * 3–4 years between events * Bulbar symptoms * Return to normal strength * Lack of continuous treatment * < 4 weeks to symptom peak * Weakness in at least 1 extremity * Areflexia Supportive electrodiagnostic results *	Type 2 Diabetes Mellitus (minority view) [11] * Progressive decline without asymptomatic periods Lack of bulbar symptoms Progressive worsening with each episode * Long-term treatment needed > 8 weeks to symptom peak Symmetric proximal and distal weakness Supportive electrodiagnostic results *

Key: * Present in this patient
